# Cytotoxicity assessment of LuxCreo and Nylon direct-printed photopolymers for orthodontic applications: An in vitro study

**DOI:** 10.1007/s00784-026-06906-8

**Published:** 2026-05-14

**Authors:** Al-Jewair T., Warunek S., Leyva Rodriguez D.M., Mali R., Caraballo A., Visser M.B.

**Affiliations:** 1 Department of Orthodontics, School of Dental Medicine, University at Buffalo, Buffalo, NY USA; 2 Department of Oral Biology, School of Dental Medicine, University at Buffalo, Buffalo, NY USA

**Keywords:** Cytotoxicity, Gingival fibroblast, Interleukins, Clear aligner, Direct-printed, Nylon

## Abstract

**Objectives:**

The aim of this in vitro study was to evaluate the cytotoxicity of two direct-printed photopolymers, LuxCreo and Nylon, intended for orthodontic applications.

**Methods:**

LuxCreo (LuxCreo Inc., Chicago, IL) and Nylon (EOS, Munich, Germany) direct-printed materials were compared to conventionally used orthodontic materials including polyethylene terephthalate glycol (PETG) and polymethyl methacrylate (PMMA). Human gingival fibroblasts (hGFB) were cultured in 12-well plates on sterilized material discs for 24-h, 72-h or 7-day intervals (n = 3). Following co-incubation of hGFB with the materials, an MTT assay was conducted to evaluate cell viability, an LDH assay was used to evaluate cell death, and ELISA was used to measure IL-6, IL-8, and IL-1β production.

**Results:**

Nylon significantly reduced cell metabolic activity at 24 h, 72 h, and 7 days, while LuxCreo showed a reduction only at 72 h and 7 days, compared to conventional materials. None of the materials induced a significant increase in cell death in hGFB. Elevated levels of IL-6 and IL-8 were observed only in the Nylon group. IL-1β levels were not significantly different between groups.

**Conclusion:**

Direct-printed materials reduced cell viability. While none of the materials induced cell death, Nylon did increase the pro-inflammatory cytokine response. Future studies should investigate the underlying mechanisms of cytotoxicity and pro-inflammatory responses to improve the safety and biocompatibility of orthodontic materials.

**Clinical relevance:**

The elevated pro-inflammatory cytokine response observed with direct-printed photopolymers highlight the need for clinical studies to investigate the mechanisms underlying cytotoxic and inflammatory responses. Future research should also evaluate the effects of long-term intraoral exposure and monitor relevant biomarkers during orthodontic treatment to better assess the safety and biocompatibility of these materials.

## Introduction

Synthetic polymers are ubiquitous in healthcare products and serve as essential components in a wide range of medical and dental devices. In dentistry, these acrylic-based materials are commonly used to fabricate clear aligners, retainers, temporomandibular disorder (TMD) splints, and sleep appliances. Clear aligner materials are generally fabricated from thermoplastic polyurethane using the thermoforming process on conventional or digital models [1]. Advancements in technology and research have led to the development of 3-dimensional (3D) direct-printed aligners as an alternative to the traditional thermoforming process. Direct-printed aligners are manufactured using an additive, layer-by-layer fabrication approach, whereas thermoformed aligners are produced through a subtractive process [2].

Direct-printed aligners have been shown to outperform thermoformed aligners in terms of accuracy, load resistance, and reduced deformation [2, 3]. These aligners offer enhanced geometric precision tailored to individual patient anatomy, allowing for more effective distribution of orthodontic forces in accordance with their mechanical capabilities [4]. One clear aligner system that incorporates advanced direct-printing technology is the LuxCreo ActiveMemory™ polymer (LuxCreo Inc., Chicago, IL). In 2023, LuxCreo introduced a direct-printed aligner resin that allows the fabrication of clear aligners directly from intraoral scans in approximately two hours. The system utilizes proprietary photopolymer formulations and 3D direct-printing technology to manufacture aligners without conventional thermoforming processes.

Beyond photopolymer-based materials, polyamides have also been explored for various clinical applications, including TMD and sleep appliances. Polyamides are polymers, either synthetic or natural, composed of repeating units connected through amide bonds. Among these polymers, Nylon 12 has emerged as a promising option for orthodontic applications, including Phase I orthodontic expanders, due to its superior flexural strength and elastic modulus [5].

These materials are highly regarded for their strength and formability. However, their extended intraoral use has raised concerns regarding biocompatibility [6]. Increasing evidence suggests that various polymers utilized in orthodontic appliances may leach chemical substances into the oral environment. This concern is supported by both in vitro and in vivo studies demonstrating such risks [7–12]. Specifically, Nylon 12, when used over prolonged periods, has been shown to elevate levels of pro-inflammatory cytokines, such as IL-8, released from macrophages. [13]

Current literature remains limited in assessing the cytotoxic effects of newly developed polymers. This gap is particularly evident for the recently introduced LuxCreo direct-printed clear aligner resin, which has not yet been systematically investigated. Furthermore, comparative evaluations between direct-printed photopolymers and conventional chemically cured or thermoformed plastics are still lacking [14]. This study aimed to compare the cytotoxic and inflammatory effects of various direct-printed and thermoformed orthodontic materials using human gingival fibroblasts (hGFB) as a model cell system. The null hypothesis was that there would be no difference between direct-printed materials and conventional thermoformed materials in cytotoxicity. There would also be no significant difference in inflammatory cytokines between the two material groups.

## Materials and methods

### Study design

This in vitro study investigated two direct-printed polymers: LuxCreo (LUX) ActiveMemory™ (LuxCreo Inc., Chicago, IL) and Nylon 12 (EOS, Munich, Germany), in comparison to thermoformed polyethylene terephthalate glycol-modified (PETG) (lnvisacryl™, Great Lakes Dental Technologies, Tonawanda, NY), and chemical cured polymethyl methacrylate (PMMA) (Biocryl, Great Lakes Dental Technologies, Tonawanda, NY), (Table 1). Human gingival fibroblast cells (hGFB) were cultured in 12-well plates on sterilized material discs. A 3-(4,5-dimethylthiazol-2—yl)−2,5-diphenyltetrazolium bromide MTT assay was performed to assess cell metabolic activity and lactate dehydrogenase (LDH) activity assay was used to evaluate the cytotoxic effects of the materials on hGFB at 24-h, 72-h, and 7-day time points. Levels of released cytokines was measured by Enzyme-Linked Immunosorbent Assay (ELISA) to assess inflammatory response.

**Table 1:** Material description

Category	Product	Manufacturer	Composition*	Applications
Direct-Printed Photopolymer	DCA Clear Aligner Resin	LuxCreo (Belmont, CA)	Vinyl ester urethane	Clear aligners, retainers
	Nylon 12 (PA 2200)	EOS (Munich, Germany)	Polyamide 12	Temporomandibular disorders splints, sleep appliances maxillary expanders
Chemical Cure	Biocryl	Great Lakes Dental Technologies (Tonawanda, NY)	Polymethylmethacrylate	Retainers, other dental
Thermoplastic	lnvisacryl™	Great Lakes Dental Technologies (Tonawanda, NY)	Polyethylene terephthalate glycol	Retainers, other dental

^*^ Composition as indicated in Safety Data Sheets (SDS) supplied by the distributor, Great Lakes Dental Technologies:

- Biocryl: Methyl methacrylate (MMA) is the liquid monomer which is polymerized to form the solid acrylic Polymethylmethacrylate (PMMA), marketed as Biocryl

- Invisacryl: Polyethylene Terephthalate Glycol (PETG) is a thermoformed resin marketed as Invisacryl

- Nylon 12: Polyamide 12, marketed as nylon 12

- LuxCreo DCA: The composition is proprietary and not disclosed in SDS files or other technical specifications. However, Fourier Transform Infrared (FTIR) analysis identify resins for direct-print orthodontic

aligners and other dental applications in a class composed of primarily urethane dimethacrylate (UDMA), also known as vinyl ester urethane.^1,2,3^

^*1*^*Can E, Panayi N, Polychronis G, Papageorgiou S, Zinelis S, Eliades G, *et al*. 2022. In-house 3D-printed aligners: effect of *in vivo* ageing on mechanical properties. Eur J Orthod. 44(1):51—55*

^*2*^*Bouchema T, Saunier J, Mollier L, Mauriello J, Brice, Savard B, *et al*. 2026. Comparison of three dental resins for 3D printing of orthodontic appliances: Comparison of leaching, biocompatibility, and thermo-mechanical properties after post-curing and aging. Dental Materials. 42:381—402*

^*3*^*Dantagnan CA, Babajko S, Nassif A, Porporatti A, Attal JP, Dursun E, *et al*. 2025. Biocompatibility of direct printed clear aligners: A systematic review of *in vitro* studies. International Orthodontics. 23:101,028. 10.1016/j.ortho.2025.101028*

### Material sample preparation

First, identical 3D printed models were prepared according to the specifications of the American Board of Orthodontics. Using these models, direct printed, thermoformed and chemical cure trays were prepared in accordance with manufacturers’ instructions. LUX aligners were printed using Dental Clear Aligner (DCA) resin (LuxCreo Inc., Chicago, IL) in an iLux Pro Dental Printer (LuxCreo Inc., Chicago, IL) under UV light at 385 nm. They were then placed in an iLuxWash Dental 2-step alcohol bath (LuxCreo Inc., Chicago, Il) for 8 min, baked in a Septree finishing oven (LuxCreo Inc., Chicago, IL) for 1 h at 90 ºC and post-cured in an iLuxCure Pro curing unit (LuxCreo Inc., Chicago, IL) at 385 nm for 30 min. Nylon appliances were printed using Nylon 12 polyamide resin (EOS, Munich, Germany) in a Velocis Formiga P110 printer (EOS, Munich, Germany) with a CO2 laser at 10,000 nm. Parts were allowed to cool to 60 ºC before de-powdering and polished in a Rador surface finishing system (Postprocess Technologies Inc., Buffalo, NY). Invisacryl™ PETG material was thermoformed at 220 ºC with a heating time of 30 s. and 4 bar pressure.

The thermoformed trays were produced by heating 1 mm sheets of Invisacryl™ PETG in a Biostar^R^ positive pressure thermoforming machine at 90 psi for 30 s which yielded 0.6 mm layer thicknesses at the labial surface of the maxillary central incisors. Chemical cure Biocryl trays were likewise prepared using a template with spacer to accept the viscous polymer-monomer mixed resin. For consistency, 0.6 mm printed trays were prepared for all materials. Discs with a standardized diameter of 5.0 mm were then prepared using an arch punch (Osborne & Co., Harrison, NJ) centered on the labial surfaces of the maxillary central incisors on the prepared trays. This diameter allowed for the disks to be conveniently inserted in 12-well culture plates. The samples were cleaned with 70% ethanol prior to being placed in glass vials for testing. Material preparation, disc fabrication, and surface finishing procedures were standardized across all materials and performed by a single investigator (S.W.) to ensure procedural consistency.

### Human gingival fibroblast cell culture and treatment

Discs made of different materials were placed in wells of sterile 12-well culture plates. For sterilization, 70% ethanol was added to all wells containing discs and to wells that served as controls (Sham, no treatment, and 0.1% SDS) in a laminar flow hood. Plates were kept in the hood with the lid off for 2 h under UV light to evaporate ethanol and further maintain sterility. To ensure no ethanol residue was left on the plates or material discs before adding hGFB cells, 12-well plates were maintained in a cell culture cabinet with the lid off for a minimum of 14 h. Ethanol treatment and UV exposure of control wells was performed in parallel to wells containing material discs to rule out any ethanol-related confounding effects. Sterilization of material discs using a combination of ethanol and UV light was selected based on methods reported for in vitro cell analysis studies using many aligner materials including PETG [15–17] and PMMA [18]. Primary hGFB cells previously isolated and archived were used in this study [19]. Cells (between passages 4 to 8) were routinely grown in Dulbecco’s Modified Eagle’s medium (DMEM) containing 10% FBS (Invitrogen). For assays, 100,000 hGFB cells were added to 12-well plates on top of the material discs in a total volume of 1.5 mL and grown for 24-h, 72-h, or 7-day time points.

### Cytokine measurement by ELISA

Fibroblast conditioned media was collected, centrifuged at 5000 × g for 2 min at 4 °C to remove cellular debris and then frozen at −80 °C until use. For measurement of human Interleukin-8 (hIL-8; R&D DY208), human Interleukin-6 (hIL-6; R&D DY206), and human Interleukin-1 beta (hIL-1β; R&D DY201) by ELISA, 100 μL of media was used per well in duplicate, following the manufacturer’s instructions. Results were calculated using a standard curve and quantified as changes in absorbance (450 nm), as measured with a FlexStation 3 Multi-Mode Microplate Reader.

### Measurement of gingival fibroblast cellular activity

Cell metabolic activity was measured using a MTT (3-(4,5-dimethylthiazol-2—yl)−2,5-diphenyltetrazolium bromide) assay. Sodium dodecyl sulfate (SDS, 0.1%) served as a positive control to induce cell death and a sham control was used as a baseline control for evaluating cell viability. 0.1% SDS was added to the corresponding negative control wells and left for 20 min. Following cell-material disc treatments, media was removed, cells washed with 1.5 mL PBS and replaced 300 μL of phenol-free media containing 500 μg/mL of MTT reagent in each well. After a 4-h incubation period, the MTT reagent media was removed, 400 μL of isopropyl alcohol added and shaken until the MTT precipitate dissolved, before removing the material discs. Absorbance values (490 nm) were measured using plate reader for analysis (FlexStation 3 Multi-Mode Microplate Reader).

To directly assess the cytotoxicity effect of the materials on hGFB, conditioned media (CM) containing secreted products was collected after material disc co-incubation, and the LDH Activity Assay was performed following the manufacturer's protocol (CyQUANT LDH Cytotoxicity Assay Kit [C20300]). Experimental testing was conducted by two investigators (D.M.L.R, R.M.).

### Statistical analysis

Statistical analyses were conducted using GraphPad Prism software (GraphPad). Comparisons between two groups were evaluated using either paired or unpaired t-tests, as appropriate. Normality was assessed using the Shapiro–Wilk test (significance 0.05). For normally distributed data, comparisons involving more than two groups was performed by one-way analysis of variance (ANOVA) followed by *post-hoc* Tukey’s HSD. For non-normal distributed data, the presence of outliers as the cause of non-normality was assessed. Identified outliers were excluded from the final parametric analysis for this data performed using ANOVA with Tukey’s HSD and is noted in the appropriate figure legend. If outliers were not identified as the cause of non-normality, the non-parametric Kruskal–Wallis H test was conducted to examine differences among groups with post hoc Dunn’s multiple comparisons test performed. All results were based on three to four independent experiments, with duplicate or triplicate wells in each. Power analysis of sample size (n = 3) estimates detection of 25% difference between groups at 10% variance at 95% confidence level (α = 0.05, β = 0.2, power = 0.80) [20]. Statistical significance was defined as *P* < 0.05. Error bars represent the standard error of the mean (SEM). Exploratory correlation analysis of cytokine production over time was analyzed using Pearson correlation coefficient.

## Results

### Direct printed materials reduce metabolic activity in human gingival fibroblasts

**Fig. 1:**
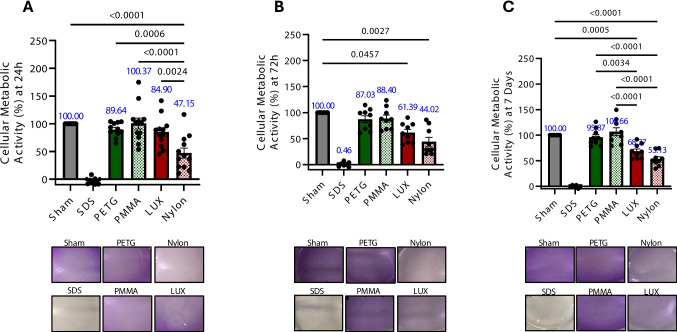
Cellular metabolic activity in human gingival fibroblasts decreases with exposure to Nylon and LUX over time. MTT assay results of human gingival fibroblasts exposed to different orthodontic materials assessing cellular metabolic activity over 24H (A), 72H (B), and 7 days (C). The images represent the MTT assay results at different time points. Representative images of MTT results for hGFB and Sodium dodecyl sulfate (SDS), as a positive control. Graphs represent the mean ± SEM, n > 3. A and C) One-way analysis of variance (ANOVA) followed by Tukey’s HSD, B) Kruskal–Wallis H test followed Dunn’s post hoc test. One outlier point responsible for non-normality was removed in data presented in Panel A

Changes in metabolic activity can lead to cellular stress, a mechanism by which cells respond to modifications in their microenvironment [21, 22]. Thus, we first began by asking whether any orthodontic material would have a considerable impact on metabolic activity in hGFB. To measure metabolic activity, the MTT assay was conducted on hGFB after 24 h, 72 h, or 7 days of exposure to the various materials. After 24 h, Nylon (direct-printed) was the only material to exhibit a considerable reduction in metabolic activity, with a significant difference from all other orthodontic materials, including sham (Fig. 1a and Table 2), as indicated by the percent change in optical density absorbance (47.15%) and represented in images, where the crystal violet color is almost comparable to that of SDS-treated cells. At 72 h (Nylon, 44.02% and LUX, 61.39%) present a significant decrease in metabolic activity compared to sham (Fig. 1b), and at 7-day time points (Nylon, 53.13% and LUX, 68.37%), direct-printed materials present a significant decrease in metabolic activity compared to sham, thermoformed, or chemically cured materials treated cells, such as PETG and PMMA, respectively (Fig. 1c). Thermoformed and chemically cured material treated groups maintained a metabolic activity similar to the sham group during the duration of treatment exposure. Overall, Nylon showed the most significant reduction in hGFB metabolic activity at 24, 72 h, and 7 days. In contrast, LUX demonstrated significant declines at 72 h compared to the sham group. After 7 days, the metabolic reduction became even more pronounced, showing significant differences when compared to the sham, PETG, and PMMA groups. This indicates that direct-printed materials influence the metabolic activity of hGFB cells.

**Table 2:** Summary of metabolic activity (MTT assay), cell membrane integrity (LDH release), IL-6, IL-1β and IL-8 results for human gingival fibroblasts exposed to PETG (C), PMMA (D), LuxCreo (E) or Nylon 12 (F) for 24 h, 72 h or 7 days. SDS exposure (B) serves as a positive control for cell death

**Biological Assay**	Sham **(A)** Mean (± SEM)	SDS **(B)** Mean (± SEM)	PETG** (C)** Mean (± SEM)	PMMA** (D)** Mean (± SEM)	LUX** (E)** Mean (± SEM)	Nylon** (F)** Mean (± SEM)	Significance (p-value comparison groups)*
**MTT Assay (%)**							
24 h	100 (0.0)	−3.401 (1.866)	89.64 (3.916)	100.4 (9.734)	84.90 (7.970)	47.15 (8.944)	< 0.0001 **A-B**, 0.8914 **A-C**, > 0.9999 **A-D**, 0.6056 **A-E**, < 0.0001 **A-F**, < 0.0001 **B-C**, < 0.0001 **B-D**, < 0.0001 **B-E**, < 0.0001 **B-F**, 0.8763 **C-D**, 0.9964 **C-E**, 0.0006 **C-F**, 0.5804 **D-E**, < 0.0001 **D-F**, 0.0024 **E–F**
72 h	100 (0.0)	0.4584 (1.633)	87.03 (6.706)	88.40 (6.833)	61.39 (6.208)	44.02 (8.585)	< 0.0001 **A-B**, > 0.9999 **A-C**, > 0.9999 **A-D**, 0.0457 **A-E**, 0.0027 **A-F**, 0.0009 **B-C**, 0.0012 **B-D**, 0.2828 **B-E**, > 0.9999 **B-F**, > 0.9999 **C-D**, > 0.9999 **C-E**, 0.1513 **C-F**, > 0.9999 **D-E**, 0.1890 **D-F**, > 0.9999 **E–F**
7 days	100 (0.0)	−0.550 (1.084)	95.87 (5.494)	106.7 (7.885)	68.37 (4.683)	53.13 (4.545)	< 0.0001 **A-B**, 0.9909 **A-C**, 0.9281 **A-D**, 0.0005 **A-E**, < 0.0001 **A-F**, < 0.0001 **B-C**, < 0.0001 **B-D**, < 0.0001 **B-E**, < 0.0001 **B-F**, 0.6315 **C-D**, 0.0034 **C-E**, < 0.0001 **C-F**, < 0.0001 **D-E**, < 0.0001 **D-F**, 0.2592 **E–F**
**LDH Assay (490—680 nm)**							
24 h	0.176 (0.038)	0.468 (0.027)	0.188 (0.042)	0.165 (0.033)	0.165 (0.024)	0.184 (0.040)	< 0.0001 **A-B**, 0.9999 **A-C**, > 0.9999 **A-D**, > 0.9999 **A-E**, > 0.9999 **A-F**, 0.0001 **B-C**, < 0.0001 **B-D**, < 0.0001 **B-E**, 0.0001 **B-F,** 0.9972 **C-D**, 0.9971 **C-E**, > 0.9999 **C-F**, > 0.9999 **D-E**, 0.9988 **D-F**, 0.9988 **E–F**
72 h	0.224 (0.031)	0.398 (0.007)	0.201 (0.020)	0.193 (0.020)	0.224 (0.023)	0.212 (0.021)	0.0002 **A-B**, 0.9787 **A-C**, 0.9299 **A-D**, > 0.9999 **A-E**, 0.9991 **A-F**, < 0.0001 **B-C**, < 0.0001 **B-D**, 0.0002 **B-E**, < 0.0001 **B-F**, 0.9999 **C-D**, 0.9791 **C-E**, 0.9992 **C-F**, 0.9309 **D-E**, 0.9913 **D-F**, 0.9991 **E–F**
7 days	0.189 (0.018)	0.475 (0.042)	0.196 (0.021)	0.181 (0.012)	0.179 (0.012)	0.190 (0.021)	< 0.0001 **A-B**, > 0.9999 **A-C**, 0.9998 **A-D**, 0.9996 **A-E**, > 0.9999 **A-F**, < 0.0001 **B-C**, < 0.0001 **B-D**, < 0.0001 **B-E**, < 0.0001 **B-F**, 0.9960 **C-D**, 0.9940 **C-E**, > 0.9999 **C-F**, > 0.9999 **D-E**, 0.9996 **D-F**, 0.9993 **E–F**
**ELISA IL-6 (pg/mL)**							
24 h	376.2 (74.08)	376.7 (64.68)	127.6 (62.82)	327.5 (76.71)	538.2 (32.88)	789.7 (200.0)	0.5116 **A-C**, 0.9993 **A-D**, 0.8607 **A-E**, 0.0670 **A-F**, 0.7228 **C-D**, 0.0702 **C-E**, 0.0008 **C-F**, 0.6773 **D-E**, 0.0306 **D-F**, 0.4989 **E–F**
72 h	264.9 (38.58)	259.3 (16.14)	225.4 (11.54)	262.4 (35.45)	428.5 (81.78)	1911 (201.0)	0.9996 **A-C**, > 0.9999 **A-D**, 0.8016 **A-E**, < 0.0001 **A-F**, 0.9997 **C-D**, 0.6242 **C-E**, < 0.0001 **C-F**, 0.7916 **D-E**, < 0.0001 **D-F**, < 0.0001 **E–F**
7 days	936.2 (71.69)	974.5 (35.82)	2834 (438.8)	1133 (85.93)	1489 (248.7)	10,492 (318.2)	< 0.0001 **A-C**, 0.9917 **A-D**, 0.5887 **A-E**, < 0.0001 **A-F**, 0.0003 **C-D**, 0.0055 **C-E**, < 0.0001 **C-F**, 0.8975 **D-E**, < 0.0001 **D-F**, < 0.0001 **E–F**
**ELISA IL-1β (pg/mL)**							
24 h	5.021 (1.605)	4.038 (1.284)	5.631 (1.337)	5.690 (0.7081)	4.569 (1.110)	5.439 (1.302)	0.9992 **A-C**, 0.9988 **A-D**, 0.9998 **A-E**, 0.9999 **A-F**, > 0.9999 **C-D**, 0.9897 **C-E**, > 0.9999 **C-F**, 0.9869 **D-E**, > 0.9999 **D-F**, 0.9959 **E–F**
72 h	0.3671 (0.151)	0.1236 (0.037)	0.4814 (0.112)	0.4309 (0.151)	0.4204 (0.108)	0.4601 (0.166)	0.9888 **A-C**, 0.9993 **A-D**, 0.9996 **A-E**, 0.9946 **A-F**, 0.9998 **C-D**, 0.9994 **C-E**, > 0.9999 **C-F**, > 0.9999 **D-E**, > 0.9999 **D-F**, > 0.9999 **E–F**
7 days	1.215 (0.098)	0.9561 (0.118)	1.224 (0.134)	1.530 (0.297)	1.399 (0.237)	1.316 (0.263)	> 0.9999 **A-C**, 0.8780 **A-D**, 0.9866 **A-E**, 0.9990 **A-F**, 0.8913 **C-D**, 0.9895 **C-E**, 0.9994 **C-F**, 0.9978 **D-E**, 0.9743 **D-F**, 0.9997 **E–F**
**ELISA IL-8 (pg/mL)**							
24 h	318.3 (70.41)	323.6 (86.65)	784.0 (205.7)	450.6 (145.4)	579.3 (141.8)	4080 (830.6)	0.9552 **A-C**, 0.9999 **A-D**, 0.9958 **A-E**, < 0.0001 **A-F**, 0.9914 **C-D**, 0.9989 **C-E**, < 0.0001 **C-F**, 0.9999 **D-**E, < 0.0001 **D-F**, < 0.0001 **E–F**
72 h	612.8 (64.94)	484.1 (35.99)	1971 (381.5)	803.9 (60.58)	1390 (457.1)	18,924 (2009)	0.0480 **A-C**, > 0.9999 **A-D**, > 0.9999 **A-E**, 0.0001 **A-F**, > 0.9999 **C-D**, > 0.9999 **C-E**, > 0.9999 **C-F**, > 0.9999 **D-E**, 0.0255 **D-F**, 0.0391 **E–F**
7 days	526.9 (58.63)	451.8 (47.73)	1702 (271.7)	693.8 (60.88)	708.3 (94.68)	22,697 (1519)	0.0953 **A-C**, > 0.9999 **A-D**, > 0.9999 **A-E**, 0.0006 **A-F**, 0.9153 **C-D**, 0.6926 **C-E**, > 0.9999 **C-F**, > 0.9999 **D-E**, 0.0228 **D-F**, 0.0144 **E–F**

^*^ Statistical significance was defined as P < 0.05

### Orthodontic materials show no cell-membrane damage cytotoxic effects on gingival fibroblast cells

**Fig. 2:**
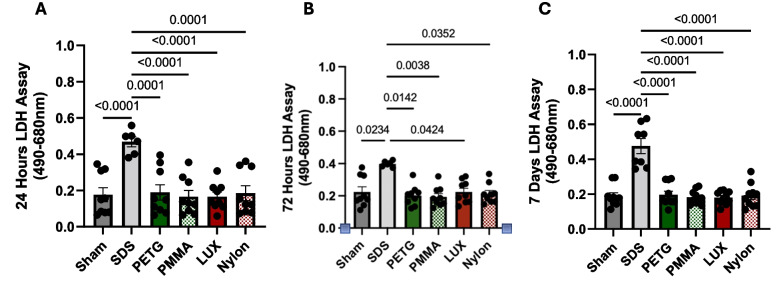
The orthodontic materials had no impact on the level of cell death in human gingival fibroblasts. LDH assay results of human gingival fibroblasts treated with different orthodontic materials assessing cellular damage over 24 h (A), 72 h (B), and 7 days (C). Sodium dodecyl sulfate (SDS) exposure served as a positive control to decrease cell activity. Graphs represent the mean ± SEM, n > 3. A and C) Kruskal–Wallis H test followed Dunn’s post hoc test, B) One-way analysis of variance (ANOVA) followed by Tukey’s HSD

After observing changes in metabolic activity in hGFB, especially during exposure to direct-printed materials, we next sought to investigate whether these differences were the result of cell membrane damage. Therefore, we measured LDH release from hGFB cells to directly assess the cytotoxicity of the various orthodontic materials. After 24, 72 h, and 7 days, no orthodontic materials showed a significant difference in LDH release among them or when compared to sham (Fig. 2a, b, and c). We also included SDS-treatment as a positive control for cell membrane damage in this assay. We observed that the SDS-treated group showed a significant increase in LDH release compared with the sham or all material-treated groups. These data suggest that direct-printed materials, such as Nylon and LUX, influence metabolic activity in hGFB cells, without inducing overt membrane rupture or necrotic cell death.

### Nylon and PETG materials led to a sustained and significantly higher secretion of IL-6 from human gingival fibroblasts

**Fig. 3:**
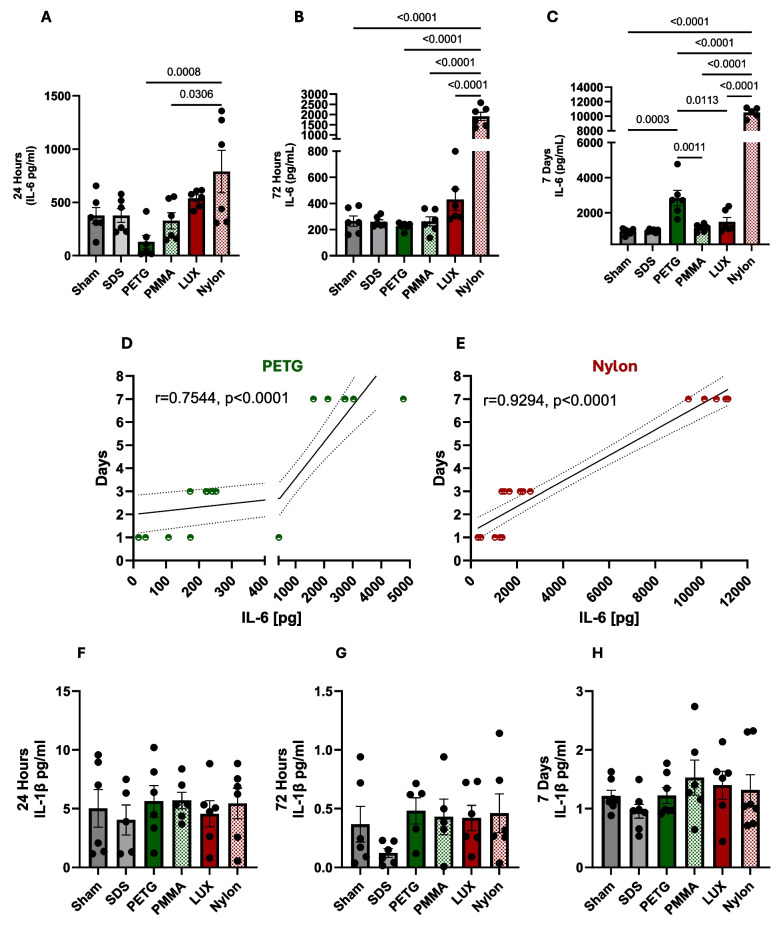
Increased IL-6 secretion in human gingival fibroblasts during exposure to PETG or Nylon materials. Human gingival fibroblasts cells were treated with orthodontic materials, SDS, or no treatment (sham). At 24 h (A), 72 h (B) and 7 days (C) secreted IL-6 was measured by ELISA. Pearson correlation analysis of IL-6 production over time for PETG (D) and Nylon (E) *r* = Pearson. Secreted IL-1β was measured by ELISA at 24 h (F) 72 h (G) and 7 days (H). Graphs represent the mean ± SEM, n > 3. One-way analysis of variance (ANOVA) followed by Tukey’s HSD

Cytokine production is a primary indicator of inflammatory response and metabolic dysfunction has been associated with changes in levels of pro-inflammatory mediators such as IL-6 and IL-1β [23–25]. As hGFB experienced a decrease in metabolic activity during direct-printed materials exposure (Fig. 1) we next evaluated how orthodontic materials would affect IL-6 secretion from hGFB via ELISA. After 24 h, Nylon, direct-printed, exhibited a considerable increase in IL-6 secretion in hGFB cells, with a significant difference when compared to PETG and PMMA (Fig. 3a and Table 2). Interestingly, neither LUX, PETG, nor PMMA had a significant effect on IL-6 secretion after 24 h of exposure when compared to the sham group. At the 72-h time point, Nylon continued to exhibit a significant increase in IL-6 secretion (Fig. 3b). In addition, at the 72-h time point, the Nylon-treated group had a significant difference in increased IL-6 levels when compared to treated groups with PETG, PMMA, Sham, or LUX. At the 7-day time point, the Nylon-treated group continued having a significant increase in IL-6 secretion in hGFB cells when compared to all other groups (Fig. 3c). Interestingly, PETG, thermoformed material, exhibited an increase of IL-6 secretion in hGFB cells at 7 days, with significant differences when compared to LUX, Sham, or PMMA, but not as robust as the Nylon-treated group (Fig. 3c).

Due to the noticeable trend in increased IL-6 secretion in hGFB cells in the PETG or Nylon treated groups, we hypothesized that IL-6 levels would correlate with the time of material exposure in hGFB cells. An exploratory Pearson correlation analysis was performed to assess the relationship between increased IL-6 over time of exposure, revealing a moderate positive correlation (r = 0.7544, p < 0.0001) between PETG and IL-6 levels (Fig. 3d) and a strong positive correlation (r = 0.9294, p < 0.0001) between Nylon and IL-6 levels (Fig. 3e). After assessing IL-6 secretion, we then asked the question whether the orthodontic materials would similarly affect IL-1β secretion in hGFB. Interestingly, after 24 h, 72 h, and 7 days of orthodontic material exposure, no significant differences were observed in either of the orthodontic material-treated groups (Fig. 3f, g, and h). Overall, these data indicate that Nylon has a robust impact on IL-6 secretion associated with changes in metabolic activity. At the same time, PETG increased IL-6 levels in hGFB cells with increasing exposure duration, without affecting metabolic activity.

### Nylon led to a sustained and significantly higher secretion of IL-8 in human gingival fibroblasts

**Fig. 4:**
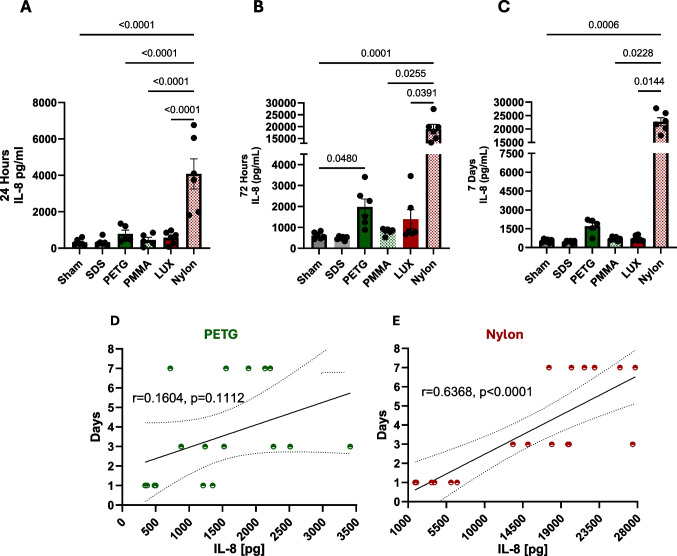
Increased IL-8 secretion in Human gingival fibroblast cells during exposure to Nylon material. Human gingival fibroblast cells were treated with orthodontic materials, SDS, or no treatment (sham). At 24 h (A), 72 h (B) and 7 days (C) secreted IL-8 was measured by ELISA. Pearson correlation analysis of IL-8 production over time for PETG (D) and Nylon (E) *r* = Pearson. Graphs represent the mean ± SEM, n > 3. A) One-way analysis of variance (ANOVA) followed by Tukey’s HSD. B and C) Kruskal–Wallis H test followed Dunn’s post hoc test. One outlier point responsible for non-normality was removed in data presented in Panel A. Table 1: Material description

Neutrophils are essential immune cells that play a significant role in modulating inflammatory responses in the oral cavity, where they represent the majority of innate immune cells [26]. Given their crucial role in promoting inflammation, we aimed to indirectly assess whether the orthodontic materials would favor a neutrophil-rich environment by measuring the secretion of interleukin 8 (IL-8), a chemokine known to recruit neutrophils [27], in hGFB cells using ELISA. After 24 h, 72 h, and 7 days, only the Nylon direct-printed material group showed a significant increase in IL-8 secretion in hGFB cells. This increase was notably greater compared to PETG, PMMA, as well as LUX and sham groups (Fig. 4a, b, and c; Table 2). Similar to IL-6 secretion levels (Fig. 3) we observed a noticeable trend of increased IL-8 secretion in hGFB cells treated with PETG or Nylon. Based on this observation, we hypothesized that IL-8 levels correlate with the duration of material exposure in hGFB cells. To evaluate this relationship, we conducted an exploratory Pearson correlation analysis between increased IL-8 levels and the duration of exposure. The results revealed a moderate positive correlation (r = 0.6368, p < 0.0001) between Nylon time exposure and IL-8 levels (Fig. 4e). In contrast, there was no correlation (r = 0.1604, p = 0.1112) between PETG time exposure and IL-8 levels (Fig. 4d). Overall, our data show that the direct-printed material, Nylon is associated with increased secretion of IL-8 from hGFB cells.

## Discussion

The evolution of intraoral devices has been marked by the development of advanced materials and innovative manufacturing techniques aimed at enhancing performance while maintaining clinical safety and minimizing biological risk. Consequently, continued research into the biological effects of both traditional and emerging materials remains essential. However, to date, no studies have reported a cytotoxicity evaluation of new direct-printed materials such as LUX, nor have they compared these with conventional chemically cured or thermoplastic polymers.

Over the past decade, the adoption of Computer-Aided Design (CAD), Computer-Aided Manufacturing (CAM), and Additive Manufacturing (AM) technologies has expanded rapidly within dental applications. Recently, direct-printed aligners have emerged, utilizing urethane-based photopolymers printed in 50 µm layers, thereby eliminating the need for intermediate physical models.

LUX DCA material is a custom-formulated, light-activated resin composed of multiple elements and designed to exhibit shape-memory behavior. Central to this formulation is LUX’s ActiveMemory™ polymer technology, which enables the aligner’s original geometry to be encoded during curing and later expressed in use. Evidence on the efficacy of LUX is still limited. However, a recent in vitro investigation reported that LUX DCA exhibited enhanced shape stability, more consistent force delivery over time, and a decreased need for auxiliary attachments as compared to conventional thermoplastic materials. [28]

Nylon is one of the most widely used industrial polymers. Nylon 6 and Nylon 12 are commonly utilized in engineering, medical, and increasingly dental applications. These materials differ significantly in structure and performance. Nylon 6 exhibits higher moisture absorption, which may compromise dimensional stability, whereas Nylon 12 offers greater flexibility and impact resistance [29]. As a result, Nylon 12 is particularly well suited for dental applications such as orthodontic appliances and sleep-related oral devices. [29]

The present study evaluated hGFB metabolic activity following 24 h, 72 h, and 7 days of exposure to different orthodontic materials, revealing that direct-printed materials significantly reduce metabolic activity in hGFB. Among the tested materials, Nylon 12 produced the most pronounced reduction at all time points (24, 72 h, and 7 days), whereas LUX caused significant decreases at 72 h and 7 days compared with PETG, PMMA, and the sham control. However, none of the materials exhibited overt cytotoxic effects as measured by membrane permeability on gingival fibroblast cells, either within the material groups or in comparison with the sham control. Consequently, the hypothesis that direct-printed materials differ from control groups in cytotoxicity was not supported.

These results contradict the findings from a recent systematic review assessing the biocompatibility of direct-printed aligners relative to thermoformed aligners [30]. The review reported higher cytotoxicity in direct-printed aligners but no release of bisphenol-A (BPA). Instead, urethane dimethacrylate (UDMA) leaching was consistently detected across studies, highlighting the need for further research into the potential health implications of UDMA release. However, none of the studies included in the review examined LUX or Nylon 12 specifically. Considerable variability was also noted across studies in terms of follow-up durations (ranging from 24 h to 14 days) and the type of fibroblast model used (mouse vs. human gingival fibroblasts).

Removable retainers used to preserve alignment of the dentition following orthodontic treatment are typically comprised of thermoformed PETG or chemical-cured PMMA. Thermoformed retainers offer esthetic, fabrication and cost advantages while chemical-cure Hawley-type alternatives have enhanced durability with less esthetic inclusion of wires on the labial surfaces of teeth. These heat-cure (PETG after thermoforming) and chemical-cure (PMMA) control materials were chosen for this study because of their different methods of processing, the amount of residual monomer, and the filler. There are also overlapping indications for removable retainers, but few studies have evaluated the biocompatibility of retainers. Previous studies focusing exclusively on thermoformed materials have also reported varying degrees of cytotoxicity. Lo et al. evaluated the viability of human periodontal ligament cells following exposure to several clear aligner materials, including PETG, thermoplastic polyurethane (TPU), and copolyester polyethylene terephthalate (PET) [16]. Their findings indicated that PETG, particularly after the thermoforming process, tended to reduce cell viability, whereas TPU and PET consistently maintained viability levels above 70%, suggesting only slight cytotoxic effects. Similarly, another study examined human gingival fibroblasts exposed to PETG and TPU and reported, through MTT analysis, that both materials exhibited mild cytotoxicity, with PETG showing increased cytotoxicity after thermoforming [7]. In line with these findings, a systematic review by Ferreira et al. concluded that most clear aligner materials are generally biocompatible but that some demonstrate mild toxicity, indicating the need for continued research and material refinement to ensure safety and long-term biocompatibility [31].

Thermoplastics such as PETG are prone to mechanical degradation in the oral environment, which may lead to the release of microplastics that could affect soft tissue health [32]. Additionally, a recent study has demonstrated that thermoformed clear orthodontic appliances used in the retention phase release more BPA in the saliva of patients when compared to the use of the classic Hawley-type appliances [33]. BPA is a monomer that used across several orthodontic and dental materials including thermoformed aligners, dental resin composites, and esthetic brackets [33]. Other studies have demonstrated that the materials composing clear aligners such as TPU can release BPA in response to the oral environment which have been demonstrated to have estrogenic effects on the body [34]. The current study did not evaluate microplastic or BPA release among the different material group, and thus a direct comparison cannot be made.

In this study, Nylon 12 and PETG materials induced a sustained and significantly elevated secretion of IL-6 in hGFB at the 7-day time point, rejecting the hypothesis of no difference in inflammatory cytokines between the two material groups. The PETG findings partially align with those of Altindal et al., who investigated short-term changes in IL-6 and IL-8 levels in the gingival crevicular fluid (GCF) of 15 individuals undergoing orthodontic treatment with thermoformed clear aligners (specifically Invisalign) [35]. Their assessments at 1 h, 3 days, 7 days, 14 days, and 21 days showed that IL-6 levels were significantly higher on days 14 and 21 compared with baseline. They also observed significant differences in IL-8 levels between baseline, days 3 and 7, and day 21. In contrast, the present study found no significant IL-8 changes for PETG or PMMA, but Nylon produced an elevated IL-8 secretion in hGFB. Overall, these findings support previous work suggesting that orthodontic aligner materials may elicit immune or cytotoxic responses capable of triggering mediator release and cellular changes.

To our knowledge, only one study has evaluated the cytotoxicity of Nylon [36]. In that study, the authors compared cell number and viability after culturing human fibroblasts with PA6 Nylon, fiber-reinforced PA6 Nylon, milled Polyether Ether Ketone (PEEK), and a no-material control at 3, 6, and 10 days. The authors reported that PEEK was biocompatible, whereas both PA6 Nylon and fiber-reinforced PA6 Nylon showed increased in vitro cell death at 10 days, suggesting that these materials may not be suitable for intraoral use beyond 6 days. These results differ from the present study, in which Nylon 12 and LUX influenced metabolic activity in hGFB cells but did not induce a significant cytotoxic effect. The discrepancy between the two studies may stem from differences in culture duration as well as differences in the chemical composition of the Nylon materials tested. Nylon 6 is a polyamide derived from caprolactam characterized by a semi-crystalline structure, whereas Nylon 12 is a polyamide based on laurolactam and is predominantly amorphous, with broader dental and orthodontic applications, including sleep appliances and TMJ devices.

Reduced metabolic activity following exposure to some aligner materials without changes in LDH activity, as observed in our study suggest sublethal cellular stress rather than overt loss of membrane integrity. The MTT assay used as a readout of metabolic activity in this study primarily measure mitochondrial reductase activity which is associated with cellular metabolic function and mitochondrial integrity. Reduced metabolic activity without membrane cell damage could be the result of cytostatic effects due to suppression of cell proliferation, mitochondrial dysfunction leading to impaired metabolic activity, early apoptotic signaling or increased oxidative stress. Alterations in cell cycle and apoptotic-related cell changes have been observed for a variety of commercial thermoformed clear aligner materials when exposed to different cell lines [37] while methacrylate-based resins increased intracellular oxidative stress and decreased cell proliferation in gingival fibroblasts [38]. Future studies are needed to investigate specific mechanisms which may be impacted by the materials used in this study; in particular Nylon-12 which demonstrated significant reduction in gingival fibroblast metabolic activity. Nylon’s inflammatory signaling may be associated with microplastic shedding as reported in a recent study [39], surface degradation or leaching of toxic components.

Clear aligners are typically worn almost full-time (approximately 22 h per day), except during eating and oral hygiene procedures, and are replaced every two weeks throughout treatment. Throughout this prolonged period, aligners remain in continuous contact with the oral environment and are exposed to factors such as temperature fluctuations, moisture, bacterial activity, and salivary enzymes [8]. They also experience mechanical stress from normal oral functions such as chewing and abrasion, as well as from parafunctional habits like bruxism [9]. Because this study was conducted under in vitro conditions, these complex intraoral factors could not be replicated or evaluated. Nonetheless, an advantage of the in vitro approach is the absence of confounding inflammatory responses associated with orthodontic tooth movement or periodontal disease. Several studies investigating biomarker levels associated with orthodontic tooth movement using clear aligners have reported increased cytokine expression at the 24-h time point [40, 41]. However, based on these findings, it remains difficult to distinguish whether the observed increase in cytokine levels is attributed to the aligner material itself or the biological cascade initiated by orthodontic tooth movement. Future research should therefore include in vivo clinical trials to better simulate clinical conditions.

This study did not assess the potential estrogenic effects of direct-printed aligners. Prastini et al. previously examined this parameter and found no estrogenic activity associated with TC-85 DAC (Graphy) [14]. Further studies should explore this outcome in other direct-printed materials to fully understand their biocompatibility and potential endocrine-related effects. Additionally, only one cell type (hGFB) was investigated, which may limit broader biological conclusions. All results were based on three to four independent experiments. A sample size of three biological replicates may represent a limitation of this study. Any significant effects observed in this study will benefit from more detailed follow-up with larger sample size. Finally, the chemical analysis of material eluates was not performed, and residual monomer release was not measured in this study. Since post-curing conditions may affect residual monomer content and influence biological responses, future studies should consider quantifying residual monomer levels to better understand how post-processing conditions may affect the observed cellular outcomes.

## Conclusion

- The metabolic activity in hGFB cells is affected during LUX and Nylon 12 exposure, indicating changes in the metabolic network but not a direct correlation to cellular membrane damage as demonstrated by LDH assay. Elevated levels of IL-6 and IL-8 were observed in the Nylon group.
- Although PETG materials did not significantly affect metabolic activity nor promote cellular damage, we observed that this material, over a prolonged period of exposure, promoted an inflammatory environment in hGFB cells, as demonstrated by an increase in IL-6 secretion.
- Within the limitations of this in vitro model and small sample size used, PMMA did not demonstrate significant effects on metabolic activity or inflammatory cytokine release.

## Data Availability

The datasets used and/or analyzed during the current study are available from the corresponding author on reasonable request.

## Declarations

We confirm that neither the manuscript nor any parts of its content are currently under consideration or published in another journal. All authors have approved the manuscript and agree with its submission.
